# Supplementary material for: End Sequence Analysis Toolkit (ESAT) expands the extractable information from single-cell RNA-seq data
Source: Genome Res. 2016 Oct;26(10):1397–410. doi: 10.1101/gr.207902.116 (PMC5052061; doi:10.1101/gr.207902.116)
Supplement: Supplemental Material [file supp_gr.207902.116_Supp_Materials.tar.gz › ESAT-esat_v0.1/doc/html/classumms_1_1core_1_1scripture_1_1_oriented_chromosome_transcript_graph.html]

ESAT: umms.core.scripture.OrientedChromosomeTranscriptGraph Class Reference


ESAT


- Main Page
- Related Pages
- Packages
- Classes
- Files

- Class List
- Class Index
- Class Hierarchy
- Class Members

All Classes Namespaces Files Functions Variables Enumerator Pages

- umms
- core
- scripture
- OrientedChromosomeTranscriptGraph

Classes |
Public Member Functions |
Static Public Member Functions |
Protected Member Functions |
List of all members

umms.core.scripture.OrientedChromosomeTranscriptGraph Class Reference

Inheritance diagram for umms.core.scripture.OrientedChromosomeTranscriptGraph:

[legend]

Collaboration diagram for umms.core.scripture.OrientedChromosomeTranscriptGraph:

[legend]

|  |  |
| --- | --- |
| Classes | |
| class | **TranscriptGraphEdge** |
|  | |
| class | **TranscriptGraphEdgeFactory** |
|  | |

|  |  |
| --- | --- |
| Public Member Functions | |
|  | OrientedChromosomeTranscriptGraph (String name, String orientation) |
|  | |
| TranscriptGraphEdge | addEdge (Annotation v1, Annotation v2) |
|  | |
| boolean | addAnnotationToGraph (Annotation a) |
|  | |
| boolean | addVertex (Annotation v) |
|  | |
| int | connectVertexToGraph (Annotation v) |
|  | |
| TranscriptGraphEdge | addEdge (Annotation v1, Annotation v2, boolean augmentCount) |
|  | |
| List< GraphPath< Annotation,   TranscriptGraphEdge > > | getPaths () |
|  | |
| Collection< Annotation > | getSourceVertices () |
|  | |
| Collection< Annotation > | getSinkVertices () |
|  | |
| Collection< Annotation > | getOrphanVertices () |
|  | |
| void | writeGraph (String save, Alignments region, boolean setUpShortExonLabels, String graphName) throws IOException |
|  | |
| VertexPair< Annotation > | getNodePair (TranscriptGraphEdge edge) |
|  | |
| Collection< GraphPath  < Annotation,   TranscriptGraphEdge > > | getOrphanPaths () |
|  | |

|  |  |
| --- | --- |
| Static Public Member Functions | |
| static Gene | pathToGene (GraphPath< Annotation, TranscriptGraphEdge > gp) |
|  | |

|  |  |
| --- | --- |
| Protected Member Functions | |
|  | OrientedChromosomeTranscriptGraph (EdgeFactory< Annotation, TranscriptGraphEdge > edgeFactory) |
|  | |

## Constructor & Destructor Documentation

|  |  |  |  |  |  |
| --- | --- | --- | --- | --- | --- |
| umms.core.scripture.OrientedChromosomeTranscriptGraph.OrientedChromosomeTranscriptGraph | ( | EdgeFactory< Annotation, TranscriptGraphEdge > | *edgeFactory* | ) |  |

protected

|  |  |  |  |
| --- | --- | --- | --- |
| umms.core.scripture.OrientedChromosomeTranscriptGraph.OrientedChromosomeTranscriptGraph | ( | String | *name*, |
|  |  | String | *orientation* |
|  | ) |  |  |

## Member Function Documentation

|  |  |  |  |  |  |
| --- | --- | --- | --- | --- | --- |
| boolean umms.core.scripture.OrientedChromosomeTranscriptGraph.addAnnotationToGraph | ( | Annotation | *a* | ) |  |

Here is the call graph for this function:

|  |  |  |  |
| --- | --- | --- | --- |
| TranscriptGraphEdge umms.core.scripture.OrientedChromosomeTranscriptGraph.addEdge | ( | Annotation | *v1*, |
|  |  | Annotation | *v2* |
|  | ) |  |  |

Here is the caller graph for this function:

|  |  |  |  |
| --- | --- | --- | --- |
| TranscriptGraphEdge umms.core.scripture.OrientedChromosomeTranscriptGraph.addEdge | ( | Annotation | *v1*, |
|  |  | Annotation | *v2*, |
|  |  | boolean | *augmentCount* |
|  | ) |  |  |

Here is the call graph for this function:

|  |  |  |  |  |  |
| --- | --- | --- | --- | --- | --- |
| boolean umms.core.scripture.OrientedChromosomeTranscriptGraph.addVertex | ( | Annotation | *v* | ) |  |

Here is the call graph for this function:

Here is the caller graph for this function:

|  |  |  |  |  |  |
| --- | --- | --- | --- | --- | --- |
| int umms.core.scripture.OrientedChromosomeTranscriptGraph.connectVertexToGraph | ( | Annotation | *v* | ) |  |

Adds an exon to a graph

Parameters
:   |  |  |
    | --- | --- |
    | v |  |

Returns

Here is the call graph for this function:

|  |  |  |  |  |  |
| --- | --- | --- | --- | --- | --- |
| VertexPair<Annotation> umms.core.scripture.OrientedChromosomeTranscriptGraph.getNodePair | ( | TranscriptGraphEdge | *edge* | ) |  |

Returns the pair of vertices for a specified edge

Parameters
:   |  |  |
    | --- | --- |
    | edge |  |

Returns

Here is the caller graph for this function:

|  |  |  |  |  |
| --- | --- | --- | --- | --- |
| Collection<GraphPath<Annotation, TranscriptGraphEdge> > umms.core.scripture.OrientedChromosomeTranscriptGraph.getOrphanPaths | ( |  | ) |  |

Returns all orphan paths in graph (self edges for orphan vertices)

Returns

Here is the call graph for this function:

|  |  |  |  |  |
| --- | --- | --- | --- | --- |
| Collection<Annotation> umms.core.scripture.OrientedChromosomeTranscriptGraph.getOrphanVertices | ( |  | ) |  |

Returns a collection of all orphan vertices.

Returns
:   All vertices having allowed edge types.

Here is the caller graph for this function:

|  |  |  |  |  |
| --- | --- | --- | --- | --- |
| List<GraphPath<Annotation, TranscriptGraphEdge> > umms.core.scripture.OrientedChromosomeTranscriptGraph.getPaths | ( |  | ) |  |

Here is the call graph for this function:

|  |  |  |  |  |
| --- | --- | --- | --- | --- |
| Collection<Annotation> umms.core.scripture.OrientedChromosomeTranscriptGraph.getSinkVertices | ( |  | ) |  |

Returns a collection of all the possible target/end vertices for this graph.

Returns
:   All vertices having allowed edge types.

|  |  |  |  |  |
| --- | --- | --- | --- | --- |
| Collection<Annotation> umms.core.scripture.OrientedChromosomeTranscriptGraph.getSourceVertices | ( |  | ) |  |

Returns a collection of all the possible source/start vertices for the specified graph.

Returns
:   All vertices having allowed edge types.

Here is the caller graph for this function:

|  |  |  |  |  |  |
| --- | --- | --- | --- | --- | --- |
| static Gene umms.core.scripture.OrientedChromosomeTranscriptGraph.pathToGene | ( | GraphPath< Annotation, TranscriptGraphEdge > | *gp* | ) |  |

static

|  |  |  |  |
| --- | --- | --- | --- |
| void umms.core.scripture.OrientedChromosomeTranscriptGraph.writeGraph | ( | String | *save*, |
|  |  | Alignments | *region*, |
|  |  | boolean | *setUpShortExonLabels*, |
|  |  | String | *graphName* |
|  | ) |  | throws IOException |

Writes the graph to a file

Parameters
:   |  |  |
    | --- | --- |
    | save |  |
    | region |  |
    | setUpShortExonLabels |  |
    | graphName |  |

Exceptions
:   |  |  |
    | --- | --- |
    | IOException |  |

Here is the call graph for this function:

---

The documentation for this class was generated from the following file:

- java/umms/core/scripture/OrientedChromosomeTranscriptGraph.java


---

Generated on Fri Aug 15 2014 15:11:15 for ESAT by  

 1.8.7
